# The Impact of Embedded Counseling Services on Mental Health Service Utilization – Data from one U.S. Public University

**DOI:** 10.1192/j.eurpsy.2026.10764

**Published:** 2026-07-31

**Authors:** R. Farah, N. Ruzek, R. L. Ferrara, K. Laczkovich, C. P. Holstege

**Affiliations:** 1 Emergency Medicine; 2 Student Health and Wellness; 3 School of Arts and Sciences, University of Virginia, Charlottesville, United States

## Abstract

**Introduction:**

Collegiate students are increasingly utilizing mental health services. University counseling centers have implemented multiple models to deliver care and expanded services. A novel model recently employed involves embedding university counseling services directly in campus academic departments in addition to the existing site of the university central counseling center. In 2020, a large public university expanded mental health services to include counseling services embedded in three academic departments.

**Objectives:**

The study’s aim is to describe changes in service utilization after embedded counseling services were initiated. We hypothesized that the availability of embedded counselors in academic departments would increase students’ access to, and subsequent utilization of, mental health services.

**Methods:**

The current study was based on two cohorts of students from a U.S. public university who used the university’s Counseling and Psychological Services (CAPS). The two cohorts included students enrolled in three schools 1) Architecture, 2) Commerce and 3) Leadership and Public Policy, between July 1, 2016 and June 30^th^, 2019 (when there were no embedded services), and July 1, 2021 and June 30^th^, 2024 (after embedded services were initiated). Data was extracted from the Student Health Research Database, an IRB-approved database connecting student data from different datasets. The study compared overall mental health utilization before and after embedded counseling was available using Generalized Estimating Equations (GEE) and robust Poisson regression models with the natural logarithm of student enrollment as offset. Rate ratios (RR) with 95% confidence intervals (CI) were computed. Data analysis was performed using R 4.4.2

**Results:**

During the 2016-2019 study period, a total of 3,378 visits to CAPS were reported. During the 2021-2024 study period, central visits accounted for 4,564 visits while embedded counseling accounted for 3,919 visits. The rates of mental health visits more than doubled (RR=2.2; 95%CI 1.9-2.6) following the initiation of embedded counseling services within the School of Architecture and within the School of Leadership and Public Policy (RR= 2.6; 95%CI 1.5-3.4), while it increased by 60% within the School of Commerce (RR=1.6, 95% CI 1.3-2.0). The rate of utilization by unique students increased 48% within the School of Architecture (RR=1.48; 95%CI 1.5-3.4).

**Conclusions:**

As the need for mental health services increases among the collegiate population, diversifying service access increases student utilization. Embedded services in addition to central services can decrease barriers and increase utilization of university mental health services.

**Disclosure of Interest:**

None Declared

